# Nonhuman Primate Models of Type 1 Diabetes Mellitus for Islet Transplantation

**DOI:** 10.1155/2014/785948

**Published:** 2014-10-20

**Authors:** Haitao Zhu, Liang Yu, Yayi He, Bo Wang

**Affiliations:** ^1^Department of Hepatobiliary Surgery, First Affiliated Hospital, Medical College, Xi'an Jiaotong University, Xi'an 710061, China; ^2^Department of Endocrinology, First Affiliated Hospital, Medical College, Xi'an Jiaotong University, Xi'an 710061, China

## Abstract

Islet transplantation is an attractive treatment of type 1 diabetes mellitus (T1DM). Animal models of diabetes mellitus (DM) contribute a lot to the experimental studies of islet transplantation and to evaluations of isolated islet grafts for future clinical applications. Diabetic nonhuman primates (NHPs) represent the suitable models of DMs to better evaluate the effectiveness of islet transplantation, to assess new strategies for controlling blood glucose (BG), relieving immune rejection, or prolonging islet survival, and eventually to translate the preclinical data into tangible clinical practice. This review introduces some NHP models of DM, clarifies why and how the models should be used, and elucidates the usefulness and limitations of the models in islet transplantation.

## 1. Introduction

Type 1 diabetes mellitus (T1DM), once known as insulin-dependent diabetes mellitus (IDDM), is an autoimmune disorder caused by progressive destruction of insulin-producing pancreatic *β*-cell that results in hyperglycemia [1, 2]. Curing patients with T1DM requires both ablation of *β*-cell-specific autoimmune reaction and *β*-cell replacement therapy [3]. At present, pancreatic islet transplantation is a promising and minimally invasive treatment that has the great potential to restore normoglycemia and achieve complete independence from exogenous insulin in T1DM patients [3–6]. Since Ballinger's and colleagues first demonstrated that islet cells isolated from rat pancreata could be transplanted into a diabetic rat model to reverse hyperglycemia, scholars pursued efforts to develop this treatment for clinical application [7, 8]. In the year 2000, by using the “Edmonton Protocol” based on a glucocorticoid-free immunosuppressive regimen, Shaprio et al. achieved insulin independence with an excellent metabolic control in diabetic patients after allotransplantation of islet mass [9]. However, due to shortage of human donors, another islet source such as from xenogenic pig or stem-cell derived functional islet emerges as an alternative strategy for transplantation [10]. Recently, several trials demonstrated the possibility of achieving sustained survival of xenogenic islet grafts in diabetic rodents or primates [11–16]; and stem-cell therapy also promised a reproducible and nearly unlimited supply of transplantable islets [17–19]. All these steady and great progresses made in the islet transplantation field likely depend on improvements in techniques for high-quality islet preparation [20–23], strategies for immune suppression or tolerance [5, 24, 25], and protocols for differentiation of stem-cell into functional *β*-cell [26]. However, animal models of diabetes mellitus (DM) also contribute a lot to islet research and to evaluation of isolated islet grafts for clinical applications [27, 28]. Moreover, to better investigate the achieved findings and to assess new protocols for relieving immune rejection and drugs/devices for prolonging islet survival, experimental research in appropriate animal models is essential. Hence, rigorous trial test of the islet grafts from various sources on preclinical large animal models of DM represents an important step to better verify the effectiveness of islet transplantation and develop novel approaches for T1DM treatment.

In large animals, diabetic nonhuman primates (NHPs) are considered as the good models for translating obtained islet transplant research safely into tangible clinical applications due to advantages including (1) evolutionary proximity to human beings, with approximately 95% of homology at the nucleotide level [29]; (2) similar clinical features of diabetes between NHPs and humans [30–33]; (3) the fact that large body size and long lifespan of NPHs make it possible and convenient to perform longitudinal studies and numerous procedures (e.g., biopsy of pancreatic tissues, extraction of large volumes of blood samples, or surgical implantation of catheters) [34]; and the fact that (4) immune system of NHPs closely resembles that of humans, making it possible and useful to study immunological aspects related with islet transplantation, as well as to develop and verify strategies to improve islet graft survival for future clinical practice. Besides, another important requirement for preclinical test in NHPs is based on the indefinite translation of rodent protocols to outbred NHPs. Even though numerous protocols are effectively applied to induce immune tolerance in rodent models, only a small amount of strategies can prolong rejection-free interval (RFI) survival without sustained severe immunosuppressive treatment in NHP models [35].

Currently, the commonly used NHP animals are baboons and macaques (rhesus and cynomolgus monkeys). Nevertheless, there are persistent hurdles to the advancement and large-scale application of these models in islet transplantation. In this review, we introduce some NHP models of DM, clarify why and how the models should be used, and elucidate the usefulness and limitations of the models in islet transplantation.

## 2. Housing and Feeding of NHPs

For promoting animal welfare and improving reliability and quality of research data in islet transplantation, academics recommend adaptations of housing and nursing care of NHP models of DM in the experimental animal center or laboratory [36]. Prior to experimental process, NHPs should be socially kept (if possible, that is, in a naturally composed breeding group, or in a peer group or same-sex group, or as a pair) in enriched laboratory conditions with (1) enhanced housing (a cage with a perch and bedding, with minimal height of 1.50 m for monkeys and 2.50 m for chimpanzees), (2) controlled temperature and light-dark cycle, (3) regular standard chow (at least twice a day) and supplementary feed (e.g., fresh vegetables and fruits; daily or weekly), (4) documented health evaluation, and (5) positive reinforcement training [37–39]. Through training, the diabetic NHPs may be able to offer a body part for blood glucose monitoring, subcutaneous or intramuscular injection, and blood collection; some primates may cooperate with intragastric administration of immunosuppressants after islet transplantation.

## 3. Species Selection

Macaque monkeys, especially cynomolgus monkey (*Macaca fascicularis*) and rhesus macaque (*Macaca mulatta*), have become the major species of choice for DM induction in the field of preclinical islet transplantation [25, 40–42] with several superior characteristics of relatively easy upkeep in captivity, wide availability, polymorphism of the major histocompatibility complex (MHC), anatomical and physiological similarity of humans, and resemblance in immune systems [34, 43–45]. More importantly, these species are usually smaller than baboons and chimpanzees, only 5–9 kilograms of males and 3–6 kilograms of females [37], and therefore have a clear superiority of much less expensive housing, care, feeding, and charges for immunosuppresive regimens. Besides that, fewer islet grafts are required to be implanted to achieve insulin independence in these diabetic recipients. Baboons and chimpanzees, although used less often [46], are likely more robust than macaques, potentially rendering them less prone to surgical and other complications [47].

## 4. Induction of DM in NHPs for Islet Transplantation

Animal experiments have enormously contributed to the study of DM, as well as islet research and transplantation. Generally, diabetic animals are classified as spontaneous and secondary (pancreatomized, chemical-induced, or gene transfection) models. However, up to now, no exact recommendation has been reached for a standardized method of DM induction.

Because spontaneous T1DM occurs in an extremely low incidence in NHPs [30], in the area of islet transplantation, DMs are usually induced in NHPs by pancreatectomy or/and by systemic administration of streptozotocin (STZ) [25, 36, 46]. Although alloxan has been reported to chemically induce a diabetic state in NHPs [34], it is generally acknowledged that this diabetogenic agent works selectively on *β*-cells of rodents and is therefore not an available and potent inducer of DM for NHPs [48–50]. Additionally, genetically induced models of DM are mainly performed in the rodent animals, such as Ins2Akita mice [51], human islet amyloid polypeptide (hIAPP) mice [52], and humanized mice with aspects of human immune system [53]. By contrast, the application of transgenic NHPs in biomedical research is just at infancy stage [54]. Currently, Huntington's disease (HD) monkey is the first and only reported transgenic NHP model of human disease [55, 56].

In NHPs, the various modeling methods exhibit different characters in pathophysiological mechanism, progression, and complications of the DM. In particular, the severity, stability, and extent of metabolic and endocrine abnormalities vary according to the induction protocols, primate species, and individual differences in monkeys.

### 4.1. Surgical Pancreatectomy

One of the most straightforward means of examining the effect of hyperglycemia in animal is to remove the pancreas, either partially or totally [57]. Pancreatomized animals, which have more than 100 y of history, are often thought of as the earliest models of DM. In the 1880s, in order to observe the intestinal absorptive function, von Mering and his colleagues excised the pancreas of a dog, and then this animal developed symptoms of polyuria and polydipsia and was eventually diagnosed as DM [57]. The extensive studies on pancreatectomized NHPs were first conducted by Gillman et al. in the 1960s [58, 59]. After pancreatectomy, hyperglycemia (hallmark of DM), polydipsia, lipaemia, and ketonaemia occurred in the baboons (*Papio ursinus*). In general, the pancreatectomized model is considered to be and utilized as a T1DM model on account of full depletion of the islet *β* cells.

Total pancreatectomy (TP) is a commonly used procedure to induce IDDM in NHPs (e.g., cynomolgus monkey, rhesus monkey, and baboon) in the preclinical studies of islet autotransplantation, as well as allotransplantation [60–65] and xenotransplantation [66, 67]. The resected pancreas can be utilized for islet cell isolation and preparation. Compared with STZ administration, TP induces IDDM more effectively, reliably, and permanently [68]. However, the complete deficiency of both pancreatic endocrine and exocrine functions also results in high probability of hypoglycemia (blood glucose < 2.8 mmol/L), unstable glycemic control, and loss of other islet hormones [69]. In view of the pancreatic function, compensatory response, and regeneration of islet mass, more than 90% of pancreas should be removed to induce stable DM in a rhesus monkey [64, 70]. Therefore, this invasive surgical procedure is more complex and requires precise manipulation. For successfully performing TP to induce DM in NHP and enabling the recipient monkey to undergo islet transplantation safely, special attention should be paid to the following points. (1) Vital signs should be monitored and maintained in a safe range during the operations: heart rate (80–160/min), systolic blood pressure (60–120 mmHg), oxygen saturation (>96%), and body temperature (35–37°C) [62]. (2) Splenic artery and vein, portal and superior mesenteric vein, arterial arcades around the duodenum, inferior mesenteric and middle colic veins, and common bile duct must be carefully preserved [64, 68, 71]. (3) Appropriate glycemic control should be performed after TP. Generally, blood glucose (BG) levels are controlled in the 100–300 mg/dL range with exogenous insulin administration before islet implantation [60–62]. (4) Controlling the fasting BG at 5–10 mmol/L and the glycosylated hemoglobin (HbA1_C_) at 3%–6.5% is a suitable strategy to induce a lower probability of hypoglycemia [69]. (5) Intensive postoperative care and management must be carried out timely to avoid complications. (6) TP and islet implantation should not be conducted concurrently but consecutively as a 2-stage surgery. Fourteen days after TP is recommended as the optimal time for islet infusion [62].

### 4.2. STZ Administration

STZ, a naturally occurring chemical (molecular formula: C_8_H_15_N_3_O_7_, molecular weight: 265.2 g/moL) derived from the soil microbe* Streptomyces achromogenes*, has been used clinically for treating metastatic pancreatic islet cell carcinomas and used investigationally for inducing experimental DM in animals [27, 72–74]. This diabetogenic drug can inhibit insulin secretion and induce a state of IDDM through its ability to result in a selective death of pancreatic *β*-cells. The cell toxicity of STZ is mainly dependent on its methylation activities leading to DNA damages. After injection, STZ is selectively taken up by islet cells via the glucose transporter 2 (GLUT2). And then, this glucosamine-nitrosourea compound leads to DNA breaks and subsequently activates poly-ADP-ribose polymerase (PARP) that result in depletion of cellular nicotinamide adenine dinucleotide (NAD^+^) and decline of subphysiological adenosine triphosphate (ATP) [72, 75]. The loss of cellular energy stores eventually causes cell death. In addition, other cells expressing the transporter GLUT2 such as hepatocytes and renal tubular cells are also susceptible to STZ; thus, the major disadvantages of STZ are hepatotoxicity and nephrotoxicity, particularly at high dose.

Compared with TP, pharmacologically induced IDDM using STZ is far more common, more convenient, and less invasive. At present, this nonsurgical approach is widely used to induce a reproducible form of DM in NHPs [36]. This is illustrated by the reports of Emory Transplant Center, in which TP in previous studies [66, 76] was replaced by STZ injection in recent studies [13, 77]. Following STZ administration, monkeys developed IDDM within 24 h, with elevated fasting BG levels (>400 mg/dL), decreased serum C-P levels (0.01–0.6 ng/mL), and loss of body weight. Even more importantly, the diabetic monkeys could remain the diabetic state up to 1 y with persistent low C-P levels (<0.6 ng/mL), no C-P release during intravenous glucose tolerance test (IVGTT), and pancreatic histology that revealed only small numbers of insulin-staining cells [78]. Although Bottino et al. reported that, in cynomolgus monkeys, pancreas could regain endogenous C-P production after complete *β*-cell destruction by high-dose STZ injection (125–150 mg/kg body weight), it seemed to be a nongeneralizable and incidental event with only 2 out of 11 diabetic monkeys recovering *β*-cell function [79].

Usually, for successful STZ administration in experimental NPHs and for optimum results, some recommendations should be followed: (1) NPHs must be fasted overnight (8–12 h) before STZ injection. (2) Owing to STZ's instability in solution, STZ should be freshly prepared, dissolved in citrate buffer (pH 4.4–4.5), stored on ice, and administered immediately within 10 min [75, 80] (Table 1). (3) The NHPs can receive antiemetic prophylaxis about 30–60 min prior to STZ injection to suppress postinfusion nausea and vomiting [81]. (4) Since obesity is a relevant risk factor for adverse events, it is suggested to preferentially select young monkeys or adult monkeys with lower girth-to-height ratio (GHtR, obesity indicator) for DM induction. In adult monkeys with a higher than normal GHtR, researchers should lower the STZ dosage by as much as 20*℅* [81]. (5) After STZ injection, aggressive fluid administration (normal saline, dose: 20–30 mL/kg, rate: 1.0 mL/min) is helpful to reduce morbidity and eliminate mortality in diabetic monkeys [81, 82]. (6) Exogenous insulin is administrated to maintain BG level < 300 mg/dL (maximum) in diabetic monkeys before transplant and following islet graft rejection [82–86]. (7) Since cyclosporine can facilitate renal dysfunction in STZ-induced diabetic monkeys [87], an intensive and careful adverse event monitoring (AEM) should be conducted as soon as immunosuppressive treatment starts in the islet transplant recipients.

Most importantly, it is necessary to establish the optimal dose of STZ that is required for inducing irreversible and stable DM with less adverse effects before evaluating treatment strategies in islet transplantation. The diabetogenic dose depends on the animal species, age, body weight, route of administration, and nutritional status [27, 75, 88]. Presently, there is still an ongoing debate with regard to the proper dose of STZ in NHPs, and STZ administration for DM induction has plenty of potential variables (Table 1). Administration of a low-dose STZ (20–50 mg/kg body weight) was not sufficient and reliable to consistently induce complete DM (C-P negative DM) in cynomolgus monkeys [89, 90]. Higher doses of STZ (80–150 mg/kg body weight) were found to be effective and sufficient [13, 81, 84, 87, 91–93] but were associated with more systemic side effects (e.g., transient vomiting, severe hypoglycemia) and serious complications (e.g., hepatic and renal function/tissue injury), as well as higher morbidity and mortality (approximately 28.6%–100%) [69, 87, 92–94]. Koulmanda et al. [78], Tal et al. [95], Dufrane et al. [94], and Zou et al. [96] demonstrated that STZ dose of 50–70 mg/kg could induce stable IDDM in all macaques for up to 0.5–1 y without any evidence of regeneration of *β*-cell, as well as liver and kidney toxicity. On the contrary, Theriault and colleagues reported the safe and consistent induction of IDDM with a single high-dose of STZ (150 mg/kg body weight) in young cynomolgus monkeys (6–8-month-old). Moreover, Wijkstrom et al. [87] recommended that the STZ dose should depend on body surface area (BSA) (1250 mg/m^2^) rather than body weight (BW). Zou et al. [96] further demonstrated that a dose > 760 mg/m^2^ of STZ might reliably induce IDDM in juvenile cynomolgus monkeys (2-3-year-old), whereas a dose < 700 mg/m^2^ was not effective. One reason for these discrepancies is that the *β*-cells of younger monkeys may be more resistant to the toxic effect of STZ than that of older ones, and this may account for the varying SZT dosages to induce DM. Another variable that may influence the dosage is the way of STZ administration. Since STZ has a short half-life (5–15 min), higher dose of STZ is necessary if the agents cannot be administrated immediately as a bolus. In addition, the individual genetic and physiological differences, variability in handing the STZ compound (i.e., scale difference, different solvent, and degradation due to preparation time and stock condition), and environmental factors (e.g., hydration protocols and subsequent regimens) also contribute to the wide variance in dose level among different research centers.

Nowadays, in preclinical studies of islet transplantation, DM is frequently induced by Zanosar STZ (Teva Pharmaceuticals) with a relatively high dosage (125–150 mg/kg or 1250–1600 mg/m^2^, intravenous injection) in recipient macaques (weight range, 3–5 kg) [13, 77, 83–86, 97]. Zanosar STZ is a clinically used drug with greater purity and less variability and therefore has fewer adverse effects than other preparations of STZ in DM induction [91].

### 4.3. Partial Pancreatectomy with STZ Administration

Since TP causes relatively high surgical morbidity/mortality [69, 71] and high-dose (100–120 mg/kg body weight) STZ can induce hepatotoxicity (e.g., hepatic steatosis) and nephrotoxicity (e.g., severe proteinuria, glomerular damage, and acute tubular injury) in diabetic NHPs [78, 81, 91, 93], partial pancreatectomy (PP, approximately 75*℅* pancreatic tissues) combined with low-dose STZ injection (15 mg/kg body weight) (PP-STZ) is considered as an alternative method for IDDM induction [71, 93]. The procedure for PP is similar to that of TP, except that the pancreatic head and uncinate process are retained in order to prevent surgical damage to important blood vessels and bile duct system. The remaining *β*-cells are then disrupted by low-dose STZ administration to avoid liver and kidney injury in NHPs caused by high-dose STZ. Although TP can lead to much lower concentration of C-peptide (C-P) (<0.1 nmol/L) than PP-STZ, there is no significant difference between the two modeling methods in terms of BG level, HbA1_C_, and exogenous insulin dosage [69, 71]. Six months after successful induction of DM, the C-P levels in the diabetic monkeys were still <0.5 mmol/L, suggesting that the DM model induced by PP-STZ was stable and credible [71]. Moreover, the PP-STZ has other advantages including stable glycemic control, less STZ-induced organ lesion, low incidence of hypoglycemia, relatively easy surgical operation, reduced complications, favorable postoperative recovery, and no mortality. All these make PP-STZ a better modeling strategy than TP and other methods (high-dose STZ administration).

## 5. Limitations of NHP Models of DM in Islet Research

On account of similar autoimmune responses that cause islet damage/loss between spontaneous type 1 diabetic animals and human T1DM, these diabetic animals are regarded as useful and reliable models for clearly elucidating the pathogenesis of T1DM, for comprehensively analyzing the induction of immune suppression/tolerance in islet transplantation, and for effectively translating the experimental findings into clinical islet transplantation in diabetic patients. Nevertheless, there is very low incidence of spontaneous T1DM in NHPs (also lower than the incidence of spontaneous type 2 diabetes), and the time course of diabetes progression is extensive too [30]. Nowadays, the most commonly used spontaneous type 1 diabetic animals are biobreeding (BB) rat and nonobese diabetic (NOD) mice [98, 99]. The autoimmunity of NOD mice is characterized by several abnormalities in the immune system including the presence of pancreas-specific autoantibodies, autoreactive CD4^+^ and CD8^+^ T cells, and defects in T regulatory cells [100, 101], which also appear in T1DM patients and rarely exist in surgical/STZ-induced NHP models of IDDM. In order to successfully perform clinical islet transplantation, it is essential to control the allo-, xeno-, and autoimmunity. The NOD mice represent the suitable models for dissecting tolerance mechanisms and assessing influences of different types of immunity in islet transplantation [102–105]. And obtained experimental results are the important improvements and supplements to the preclinical findings in diabetic NHPs. Finally, the combined data will roundly evaluate the immunosuppressive strategies and further promote immune tolerance in the future clinical islet transplantation.

Additionally, in order to clarify and improve the translational value of preclinical trials of islet transplantation, other intrinsic limitations in the NPH models of DM should also be considered, especially in pig-to-NHPs islet xenotransplantation [106]. (1) Monkeys maintain a lower set point for normoglycemia but higher insulin and C-P secretion than pigs and humans. Given the differences in glucose metabolism, it is harder to achieve stable glucose homeostasis in pig-to-NHP islet xenotransplantation [107–109]. (2) The therapeutic window of some of the commonly used immunosuppressants (e.g., cyclosporine, tacrolimus, and rapamycin) is much smaller in NHPs than in humans [109], possibly resulting in more serious side effects and rendering the NHP models less predictive for the clinical condition. (3) Severe and progressive body weight loss occurs frequently because of gastrointestinal disturbance and diarrhea, particularly if TP is carried out to induce a stable IDDM. The nutritional deficiencies can result in susceptibility to infectious complications, as well as misconception of BG and C-P levels regarding islet graft function [109]. Addressing the problems of diabetic NHP model will eventually lead to optimal experimental designs and proper use of diabetic animals from experimental islet transplantation to translational studies.

## 6. Conclusions

In summary, NHPs represent useful and suitable diabetic animal models for evaluating the effects of therapeutic interventions on the outcome of preclinical islet transplantation. Although NHP models of DM can be induced by several methods, the revulsive mechanisms are greatly different. Therefore, in islet transplantation, it is necessary to choose a proper NHP model on the basis of the aim and translational value of the preclinical study. Moreover, further exploring and clarifying the characteristics and limitations of diabetic NHP models will significantly draw the obtained preclinical data closer to clinical trials.

## Figures and Tables

**Table 1:** DM induction in NHPs by STZ administration.

Author and publication year	Animals tested (gender, age, and BW)	STZ dose	Solvent	Route of administration	Results
Theriault et al., 1999 [82]	Cyno (6–8 months, 1.0-2.0 kg), *n* = 11	150 mg/kg	0.9% NS	via SVAP	All persistently hyperglycemic (BG > 200 mg/dL; 9/11 serum C-P < 0.5 ng/mL, 60 days; 2/11 C-P at 0.9 ng/mL, 159 days); all without any observable clinical complications except transient nausea and vomiting

Shibata et al., 2002 [92]	Rhesus (female, 2.5–7.5 years, 4.2–6.8 kg)	STZ (Upjohn) G1: 100 mg/kg (*n* = 2); G2: 125 mg/kg (*n* = 4); G3: 150 mg/kg (*n* = 7)	Citric acid (pH 4.5)	via CVC	G1: none diabetic (unchanged basal insulin, *N*_IPCs_ in islets was 7.7*℅* of normal values); G2: all diabetic (IDDM) without mortality and hepatic/renal injury (*N*_IPCs_ was 0.3*℅* of normal values); G3: 2/7 dead (28.6*℅* mortality), 5/7 diabetic (up to 3 weeks, no IPCs)

Koulmanda et al., 2003 [78]	Cyno (male, 3–6 years, 3.1–9.0 kg)	G1: 100 mg/kg (*n* = 4); G2: 55 mg/kg (*n* = 20)	0.9% NS	i.v.	All groups diabetic (IDDM, BG > 400 mg/dL, Serum C-P < 0.6 ng/mL, loss of BW, decreased *N*_IPCs_); G1: impaired liver/kidney functions, severe hepatic steatosis, and acute renal tubular injury; G2: normal liver/kidney functions, no histological changes

Tal et al., 2004 [95]	Cyno (4–6 years, *n* = 6); Rhesus (8–12 years, *n* = 3); Pigtail (2 years, *n* = 5)	STZ (Zanosar) 50–70 mg/kg	5% dextrose	Selective arterial injection	All diabetic (BG > 200 mg/dL, complete obliteration of *β*-cell, 20–270 days of duration); 1/14 dead (gastric dilatation); no abnormalities in liver and kidney

Rood et al., 2006 [91]	Cyno (male, 2-3 years, 2.7–4.1 kg)	G1: STZ (Sigma), 1250 mg/m^2^ (*n* = 4); G2: STZ (Sigma), 60 mg/kg (*n* = 1); G3: STZ (Zanosar), 150 mg/kg (*n* = 5)	NM	i.v.	G1: all completely diabetic (BG > 200 mg/dL, C-P < 0.5 ng/mL, low *N*_IPCs_), with several AEs (vomiting, protein-losing nephropathy, and low ALB levels); G2: failed to loss complete *β*-cell function (C-P at 1.86 ng/mL), no AEs; G3: all completely diabetic (BG > 200 mg/dL, C-P < 0.5 ng/mL, low or no *N*_IPCs_), with only transient vomiting

He et al., 2011 [69]	Rhesus (3–6 years)	G1: single STZ ad. (80 mg/kg), *n* = 7; G2: multiple STZ ad. (25 mg/kg for 5 consecutive days), *n* = 9	NM	i.v.	All groups diabetic; G1: lower C-P levels (<0.1 nmol/L) and probability of hypoglycemia (0.41); G2: relatively higher C-P levels (<0.5 nmol/L) and probability of hypoglycemia (0.80)

Zou et al., 2012 [96]	Cyno (male, 2.4–4.7 kg)	STZ (Sigma) G1: 100 mg/kg (*n* = 7); G2: 68 mg/kg (*n* = 6); G3: 60 mg/kg (*n* = 2)	Citric acid (pH 4.5)	i.v. (via saphenous vein)	G1 and G2: all diabetic (mean fasting BG > 22 mmol/L, low *N*_IPCs_, >8 months of duration), without any marked hepatic/renal injury; G3: one diabetic (fasting BG at 26.4 mmol/L, C-P at 28 pmol/L); the other nondiabetic (fasting BG at 5.7 mmol/L, C-P at 413 pmol/L)

Overview of published studies of STZ-induced diabetic models in NHPs.

NM: not mentioned; DM: diabetes mellitus; NHP: nonhuman primate; STZ: streptozotocin; BW: body weight; G: group; Cyno: cynomolgus monkey; Rhesus: rhesus monkey; Pigtail: pigtail macaque; NS: normal saline; SVAP: subcutaneous vascular access ports; CVC: central venous catheter; BG: blood glucose; C-P: c-peptide; IDDM: insulin-dependent diabetes mellitus; NIPCs: number of insulin-positive cells; i.v.: intravenous; ALB: albumin; AEs: adverse events; and ad.: administration.
